# Induction of hemangiosarcoma in mice after chronic treatment with S1P-modulator siponimod and its lack of relevance to rat and human

**DOI:** 10.1007/s00204-018-2189-9

**Published:** 2018-03-19

**Authors:** Francois Pognan, J. Andreas Mahl, Maria Papoutsi, David Ledieu, Marc Raccuglia, Diethilde Theil, Sarah B. Voytek, Patrick J. Devine, Katie Kubek-Luck, Natalie Claudio, Andre Cordier, Annabelle Heier, Carine Kolly, Andreas Hartmann, Salah-Dine Chibout, Page Bouchard, Christian Trendelenburg

**Affiliations:** 1 Preclinical Safety, Novartis, Basel, Switzerland; 2 Pharmacokinetics Sciences, Novartis, Basel, Switzerland; 3 Preclinical Safety, Novartis, Cambridge, MA USA; 4 Discovery Investigative Safety, Preclinical Safety, Novartis, Klybeckstrasse 141, 4057 Basel, Switzerland

**Keywords:** Siponimod, Hemangiosarcoma (HSA), Placental growth factor (PLGF2), Vascular endothelial cells, Sphingosine-1-phosphate Receptor 1 (S1P1)

## Abstract

A high incidence of hemangiosarcoma (HSA) was observed in mice treated for 2 years with siponimod, a sphingosine-1-phosphate receptor 1 (S1P1) functional antagonist, while no such tumors were observed in rats under the same treatment conditions. In 3-month rat (90 mg/kg/day) and 9-month mouse (25 and 75 mg/kg/day) in vivo mechanistic studies, vascular endothelial cell (VEC) activation was observed in both species, but VEC proliferation and persistent increases in circulating placental growth factor 2 (PLGF2) were only seen in the mouse. In mice, these effects were sustained over the 9-month study duration, while in rats increased mitotic gene expression was present at day 3 only and PLGF2 was induced only during the first week of treatment. In the mouse, the persistent VEC activation, mitosis induction, and PLGF2 stimulation likely led to sustained neo-angiogenesis which over life-long treatment may result in HSA formation. In rats, despite sustained VEC activation, the transient mitotic and PLGF2 stimuli did not result in the formation of HSA. In vitro, the mouse and rat primary endothelial cell cultures mirrored their respective in vivo findings for cell proliferation and PLGF2 release. Human VECs, like rat cells, were unresponsive to siponimod treatment with no proliferative response and no release of PLGF2 at all tested concentrations. Hence, it is suggested that the human cells also reproduce a lack of in vivo response to siponimod. In conclusion, the molecular mechanisms leading to siponimod-induced HSA in mice are considered species specific and likely irrelevant to humans.

**Electronic supplementary material:**

The online version of this article (10.1007/s00204-018-2189-9) contains supplementary material, which is available to authorized users.

## Introduction

Hemangiosarcoma (HSA) is a malignant tumor arising from anarchical proliferation of vascular endothelial cells (Carmeliet 2005) and may arise from transformation of tissue-resident endothelial cell populations, from circulating progenitors, adult stem cells recruited from bone marrow, or possibly also from extramedullary sites of hematopoiesis such as the liver and spleen (Cohen et al. 2009). HSA is extremely rare in humans with less than 0.001% affected, while in various strains of rat it is mentioned to be between 0.1 and 2% in lifetime studies (Cohen et al. 2009). In mice, the spontaneous incidence is higher in males than females and ranges from 2 to 5% (Cohen et al. 2009). In CD1 mice, the spontaneous incidence of HSA is reported to be about 3.1% on average, observed mainly in liver, spleen, subcutaneous tissue, bone marrow, and skeletal muscles (RITA 2016).

Siponimod is a dual sphingosine-1-phosphate receptor 1 (S1P1) and 5 (S1P5) selective modulator, developed for the oral treatment of multiple sclerosis (Kappos et al. 2016, 2018; Pan et al. 2013). Sphingosine-1-phosphate (S1P) is a biologically active sphingolipid, interacting with five S1P receptors with various tissue expressions and involved in a number of pathways during embryological development as well as in adult biology (Blaho and Hla 2014; Rosen et al. 2013). In particular, S1P and its receptor 1 are involved in angiogenesis during embryofetal development (Mendelson et al. 2013; Skaznik-Wikiel et al. 2006), in neo-angiogenesis during wound healing (Kawanabe et al. 2007; Lee et al. 2000), during the female estrous cycle, and during pregnancy in the uterus and ovaries (Dunlap et al. 2010; Skaznik-Wikiel et al. 2006), and in neo-angiogenesis during solid tumor development (Cuvillier et al. 2013; Kunkel et al. 2013; Pyne and Pyne 2010; Takabe and Spiegel 2014; Takuwa et al. 2010). In adults, blood vessels are normally quiescent but the VECs remain highly plastic and can readily respond to angiogenic signals. Siponimod, with an EC_50_ of 0.4 nM for S1P1 and of 1 nM for S1P5, is effectively acting as an antagonist of S1P biological activity by sequestering its receptors intra-cellularly, thus preventing activation by its natural ligand S1P. Modulation of S1P receptors results in inhibition of the egress of lymphocytes from lymph nodes and Peyer’s patches, and thereby reduces the recirculation of lymphocytes to blood and tissues including the CNS (Blaho and Hla 2014; Fyrst and Saba 2010).

After 2 years of treatment with siponimod, the percent of siponimod-treated mice affected by HSA were 67, 70, and 69% for males and 53, 49, and 56% for females at 2, 8, and 25 mg/kg/day, against 14 and 13% in controls, respectively. HSA-related premature deaths were as early as after 9-month of treatment at low and high doses with tumors found in subcutis and jejunum. There was an acceleration of such incidences from month 12 onward at all doses with the highest incidence in liver and subcutis, the most frequent primary sites of HSA resulting in premature death. Importantly, in some animals multiple tissues were affected. Tissues most commonly affected with vascular tumors were adipose tissue, subcutaneous tissue, liver, spleen, heart, skeletal muscle, bone marrow, ovaries, uterus, gastrointestinal tract, and tail. Together, subcutis and liver represented the most frequent primary sites of HSA resulting in premature death. There were no such observations in rats treated similarly for 2-years at 10, 30, 90 mg/kg/day in males and 3, 10, 30 mg/kg/day in females.

Previous comparable observations have been made for other compounds. Pregabalin, an α2δ subunit voltage-gated calcium channel modifier developed and commercialized for the treatment of neuropathic pain and epilepsy, induced HSA in liver, spleen, and bone marrow of B6C3F1 and CD1 mice, but not in rat, after 2 years of treatment (Criswell et al. 2012; Pegg et al. 2012). The Pregabalin mode of action for HSA formation in mice was proposed to be the result of prolonged hypoxia due to metabolic alkalosis. This hypoxia led to sustained production of HIF-1 (Hypoxia Induction Factor 1), a key transcription factor which controls pro-angiogenic factors (Rey and Semenza 2010). In rats however, despite an initial metabolic alkalosis, some compensatory mechanisms restored normal cellular respiration and avoided chronic hypoxia. The absence of the above key events in human under Pregabalin treatment strongly suggests that the formation of such vascular tumors induced by this drug is not relevant for man (Cohen et al. 2009; Criswell et al. 2012). Peroxisome proliferation-activated receptor gamma (PPARγ) agonists are also known to induce HSA in mice, but not in rats or in humans (Kakiuchi-Kiyota et al. 2009). A similar mode of action (MOA) has been postulated whereby initial and sustained hypoxic conditions in mice, which over time induces HSA mainly in adipose tissues (Cohen et al. 2009) where PPARγ is abundantly expressed (Braissant et al. 1996). Finally, the chemical solvent 2-butoxyethanol (2-BE) has been reported to induce HSA in the liver in male mice, but not in female mice or in rats. The MOA of this chemical also involves pro-inflammatory steps and macrophage/Kupffer cell activation as for PPAR agonists, followed by hypoxia and HIF-1 activation, leading to VECs proliferation (Cohen et al. 2009; Laifenfeld et al. 2010). Hence, for these three categories of compounds, after different initial events, hypoxia and subsequent sustained activation of HIF-1 seems to be a common mechanism leading to VEC proliferation and HSA induction in mouse, but not in rat and human (Cohen et al. 2009).

To explore the molecular mechanism of siponimod-induced HSA in mice and the reasons for the resistance to this induction in rats, once daily oral gavage investigative studies were performed in the mouse and rat for 9 and 3 months, respectively. Blood and several organs of relevance were sampled at early-, mid-, and late time points. Soluble growth factors in plasma and gene expression profile (GEP) in selected organs were analyzed for each time point. Cell proliferation induction and vascular cell activation markers were assessed by immunohistochemistry in selected organs. In parallel, mouse, rat, and human primary microvascular endothelial cells were exposed in vitro to a wide concentration range of siponimod. Cell proliferation and growth factor release into the media were monitored, allowing the comparative assessment of endothelial cell response from mouse, rat, and human cells.

## Materials and methods

### In vivo mouse and rat studies

All the studies were conducted in compliance with Animal Health regulations, in particular following the Council Directive No. 2010/63/EU of 22 September 2010 on the protection of animals used for scientific purposes.

Male CD-1 mice were treated with siponimod, suspended in aqueous 0.5% Klucel solution, by oral gavage at 25 and 75 mg/kg/day for 1, 3, 7, 14, 28, 91, and 274 days. The animals were supplied by Charles River Laboratories France, l’Arbresle, France. At the beginning of the respective treatment periods, the animals were 7–8 weeks old and had a mean body weight of 36.6 g. There were 30 animals per group for each dose and time points, except for the 274-day groups which consisted of 50 animals. These numbers were chosen to ensure a powered statistical significance, and the latest time point had more animals to take into account the higher variability of individual data with increasing age, a higher probability to identify individual animals with early occurrence of HSA and potential premature death. The lowest dose used was the same as the high dose in a previous carcinogenicity study and the high dose in the current study was threefold the latter and estimated to be the maximum tolerated dose (MTD) for this duration of administration. Groups of animals were sacrificed at several early and mid-time points in order to observe initial events and the persistence and evolution of pathways and growth factors modulated by treatment with siponimod. The late time point permitted monitoring the sustainability of observed early events and to ensure the reproducibility of HSA observations potentially linked to the observed molecular events. Several organs with high incidences of HSA (e.g., liver, subcutis, muscle) in the mouse carcinogenicity study were collected for histopathology, immunohistochemistry (IHC), in situ hybridization (ISH) and gene expression profiles (GEP), as well as blood for monitoring circulating pro- and anti-angiogenic factors.

The molecular mechanisms leading to increased incidences of HSA in the mouse study were subsequently investigated in male Wistar-Han rats using a similar design with a shorter duration since no HSA were expected, as none were observed in this species after 2 years treatment with siponimod. Rats were treated with siponimod by oral gavage at 90 mg/kg/day for 1, 3, 7, 14, 28, and 92 days. This dose corresponds to a similar siponimod systemic exposure (AUC) in mice treated at 25 mg/kg/day. The Crl:Wi(Han) rats were supplied by Charles River Laboratories France, l’Arbresle, France. At the beginning of the respective treatment periods, the animals were 8–10 weeks old and had a mean body weight of 269 g. There were 30 animals per group for each dose and time point.

### Toxicokinetic

Blood was collected in a lithium heparin tube or in Venosafe tubes containing Na-fluoride, citrate buffer, and K2-EDTA. In the mouse study, plasma specimens were obtained for each dose group at time point 5 ± 0.5 h after the administration on days 1, 91, and 274. In the rat study, plasma specimens were obtained at time point 0.5, 1, 3, 6, 8, and 24 h after dosing on days 1 and 28. All tissue samples were homogenized using the fast prep device and aliquot of the internal standard working solution was added to each sample, with the exception for the blanks. The samples were extracted using tert-butylmethyl ether and evaporated to dryness. The dry residue was reconstituted in methanol/20 mM ammonium acetate in water.

Sample extracts were analyzed by LC–MS/MS in single reaction monitoring using positive electrospray ionization (ESI) as the ionization technique. The liquid chromatography was performed on a Rheos Allegro (Flux Instruments AG, Basel, Switzerland) system. A Thermo TSQ Vantage mass spectrometer (Thermo Fisher Scientific, San Jose, CA, USA) equipped with an ESI source was used for the MS/MS detection.

### Pathology and histological localization

All tissues for microscopic examination were trimmed and embedded in paraffin wax (maximum three organs per block). Tissues for microscopic examination were sectioned at a thickness of approximately four microns and stained with hematoxylin-eosin.

Immunohistochemistry and in situ hybridization (ISH) were applied on skeletal muscle tissues from a selection of animals of the control group and after 3, 7, 14, and 28 days of treatment. The selection of skeletal muscle was based on gene expression profiling results. Immunohistochemistry staining for all selected markers was performed using the fully automated instrument Ventana Discovery XT® (Roche Diagnostics AG, Rotkreuz, Switzerland). All reagents were also provided by Roche Diagnostics. Stained tissue sections were assessed by light microscopy. Images were captured with the Hamamatsu Nanozoomer slide scanner and Zeiss AxioCam/AxioVision In situ hybridization (ISH) protocol.

ISH was performed using partially the automated instrument Ventana Discovery Ultra® (Roche Diagnostics AG, Rotkreuz, Switzerland). All reagents were provided either by Roche Diagnostics or by Advanced Cell Diagnostics. The ISH probe was purchased by Advanced Cell Diagnostics Inc (Hayward, CA, USA). Slides were placed in the Ventana Ultra instrument and started using the procedure mRNA DAB Discovery Ultra 2.0 with the predefined parameters and using the combined Ventana and ACD required kit reagents (RNAscope® VS Reagent Kit-BROWN, reference 320600 and mRNA BROWN, Amp & Pretreatment PTO kit, reference 07074654001). Counterstaining was performed using Hematoxylin II and Bluing reagent. The slides were scanned on the NanoZoomer 2,0-HT scanner instrument (Hamamazu Photonics France, Massy, France) using the ×40 objective. Automated quantitative assessment of Ki67-stain-positive nuclei and tissue areas was performed on whole slide scan data using HALO image analysis software (Version 2.0, Indica Labs, Corrales (NM), USA). For the detection of stain-positive nuclei the algorithm “CytoNuclear v1.4” has been configured and combined with the classifier.

Data analysis was performed using R Version 3.2.5 and RStudio Version 0.99.893. Analysis was scripted to produce the final data table. The scripts are stored along with the raw data.

### Gene expression profile signatures

Samples for genomics assessment were collected and stored at − 80 °C until processing. Total RNA extraction of samples was conducted at CiToxLAB (Evreux, France). Briefly, total RNA was obtained by acid guanidinium thiocyanate-phenol-dichloromethane extraction from frozen tissue and the total RNA was then purified on an affinity resin (RNeasy, Qiagen) according to the manufacturer’s instruction. Total RNA was quantified by the absorbance at 260 nm, and the quality was determined using an Agilent 2100 Bioanalyzer (Agilent Technologies). RNA was stored at approximately − 80 °C until analysis. All GeneChip experiments were conducted at CiToxLAB (Evreux, France) on the Mus musculus Mouse430_2 array platform and on the Rattus norvegicus Rat230_2 array platform (Affymetrix, Inc., Santa Clara, CA, USA). The data were checked for quality, exported to Novartis and loaded in COMPARE 4.7.4 (Novartis internal software analytical tool) for analysis. Scores were calculated as geometric mean of fold changes of all genes of a signature, comparing treatment groups to time-matched control groups.

Gene signatures were established as a set of genes with highly correlated expression profiles belonging to the same molecular pathway or cell type, and manually curated based on peer-reviewed literature analysis (Stiehl et al. 2017).

### Plasma growth factors

Mouse study: Blood was collected from the vena cava at the end of the respective treatment periods on days 1, 3, 7, 14, 28, 91, or 274 (immediately before necropsy, 5 h ± 30 min after the last treatment). The blood samples were kept on ice pending centrifugation for plasma separation within 60 min after collection at 3000×*g* for 10 min under refrigerated conditions (set to maintain at + 4 °C). Plasma concentrations of Amphiregulin, EGF, Endoglin, Endothelin-1, FGF-2, Follistatin, G-CSF, HGF, IL-1β, IL-17A, IL-6, KC/CXCL1, Leptin, MCP-1, MIP-1α, PLGF2, Prolactin, sALK-1, SDF-1, sFasL, TNFα, VEGF-A, VEGF-C, and VEGF-D were determined for all surviving animals from all groups by the Luminex® xMAP® technology using the Milliplex MAP Mouse Angiogenesis/Growth Factor Magnetic Bead Panel kit (Merck Millipore, catalogue reference: MAGPMAG-24K) with a Luminex® 200 instrument.

Rat study: In the absence of commercially available rat-specific PLGF2 assays with acceptable analytical performance, two different mouse-specific assays having shown cross-reactivity to the recombinant rat PLGF2 were used. In animals from groups 1 to 10 (animals treated for 1 day, 3 days, 1 week, 2 weeks, or 4 weeks with only terminal bleed), PLGF2 plasma concentrations were measured by the Luminex® xMAP® as mentioned for the mouse. In animals from groups 12 and 13 (animals treated for 13 weeks; repeated sublingual bleeding on day 3, day 25, and day 81; terminal bleeding on day 92), PLGF2 plasma concentrations were measured by an ELISA method using the Mouse PLGF2 Quantikine ELISA kit (R&D Systems, catalogue reference: MP200) with a VersaMax® ELISA microplate reader.

Plasma concentrations of KC/CXCL1, leptin, TNFα, and VEGF were determined for all surviving animals from groups 1 to 10 by the Luminex® xMAP® technology using the Milliplex MAP Rat Cytokine/Chemokine Magnetic Bead Panel kit (Merck Millipore, catalogue reference: RECYTMAG-65K) with a Luminex® 200 instrument.

Data from all mouse and rat groups were compared statistically using a one-way ANOVA test for overall assessment and a Sidak’s multiple comparisons test for group comparisons. *p* Value < 0.05 was considered statistically significant.

### In vitro assessment of cellular proliferation and PLGF2 secretion in primary microvascular endothelial cells

Multiple primary VEC types were used in vitro to characterize the possible increase in cell proliferation and PLGF2 secretion in response to siponimod, comparing that to concentrations which may cause cytostatic or cytotoxic effects. CD-1 mouse skeletal muscle, and Sprague-Dawley (there were no Han-Wistar available cells) rat pulmonary and dermal VECs were obtained from Cell Biologics (Chicago, IL, USA), whereas human pulmonary and dermal VECs were obtained from PromoCell (Heidelberg, Germany). These cells were expanded for 2–6 passages before being used for experiments. For testing siponimod, rodent cells were plated at 40,000 cells/well (mouse) and 20,000 cells/well (rat) in gelatin-coated 96-well plates. Human cells were plated at 15,000 cells/well in uncoated 96-well plates. Exposures were carried out for 24 h, starting the day after plating. Cells were approximately 95% confluent at the time of exposures. Concentrations of siponimod tested ranged from 0.001 to 5000 nM, with a final concentration of 0.5% dimethyl sulfoxide (DMSO). After 24 h of compound treatment, half the medium was removed and stored at − 80 °C for analysis of secreted PGLF2 levels. Cells were then labeled for active DNA synthesis by incubating at 37 °C for 3 h with 10 µM 5-ethynyl-2′-deoxyuridine (EdU), before being fixed at room temperature for 15 min with 4% paraformaldehyde and processed for staining with the Click-it EdU kit (ThermoFisher). Cells were counterstained with Hoechst (ThermoFisher) at 10 µM for 1 h. The plates were washed twice with phosphate buffered saline and scanned at 20× on a Cellomics Arrayscan instrument (ThermoFisher). Percent of EDU-labeled nuclei was determined for each condition relative to total numbers of Hoechst-stained nuclei.

Cell culture medium was evaluated for PLGF2 levels using the R&D Systems (Minneapolis, MN, USA), with rat PLGF2 levels measured using the mouse assay and including a standard curve of recombinant rat protein from PromoKine (Heidelberg, Germany).

## Results

### Toxicokinetic evaluation

In mice, all animals receiving siponimod were exposed to parent compound. The mean siponimod plasma concentrations increased with increasing dose in a roughly dose-proportional manner (Table 1). The concentrations found in tissues were notably higher than that reported in plasma in mice. In rats, all animals receiving siponimod were exposed to the parent compound. No distinct accumulation of siponimod in plasma was found over the study days. In plasma and tissues in both species, the mean parent compound concentration ranged from 4.5 µM in rat muscle to 974 µM in mouse liver (Table 1), which was considerably higher than the pharmacological dose with an IC_50_ of 0.4 nM. Hence, in both species, animals were adequately exposed to the drug and the plasma concentrations in the two exploratory studies were similar to those measured in the 2-year carcinogenicity studies in mice and rats.

**Table 1:** Mean siponimod concentrations in mouse and rat plasma and tissues concentrations at various time points

	Rat		Mouse					
	Day 1	Day 28	Day 1		Day 91		Day 274	
Dose mg/kg/day	90	90	25	75	25	75	25	75
Plasma Cmax	22.3	16.6	18.2	50.3	26.5	53.6	13.5	35.6
Liver	nd	111	nd	nd	254	974	226	960
Muscle	nd	4.5	nd	nd	134	248	114	209

Values expressed in µM (approximating 1 g of tissue to 1 mL); *n* = 2 animals for rat and *n* = 5 animals for mice

*nd* not determined

### Pathology

HSA were present in two mice out of 43 sacrificed at the last time point after 9-months of dosing siponimod at 75 mg/kg/day. One affected animal presented a macroscopic mass in the papillary process of the liver which correlated microscopically with a HSA of approximately 0.5 cm in diameter. The other HSA-affected animal had a right testis enlarged by a white mass of 0.2 cm in diameter which correlated microscopically with a poorly demarcated neoplastic mass consistent with HSA. No further neoplastic or pre-neoplastic lesions were noted in any other organs evaluated in these two animals. Histopathology was not assessed in the rat study as no HSA were expected and no gross lesions were observed in this species.

### Gene expression profile signatures

**Fig. 1:**
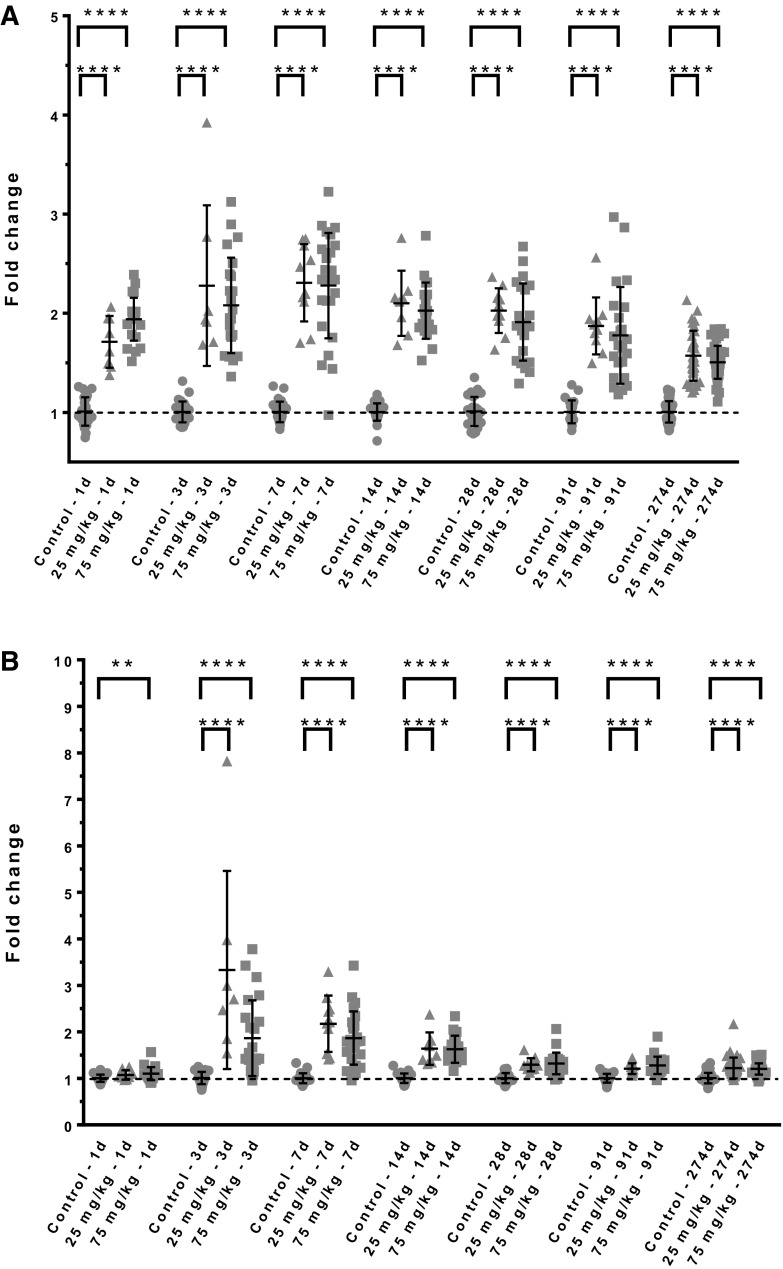
Mouse skeletal muscle VEC gene activation expression. Circle: control, triangle: low dose, square: high dose. Each dot represents the gene signature of 1 animal. **p < 0.01; ****p < 0.0001 (homoscedastic *t* test). Dotted line represents the average of control values normalized to 1 from which fold changes were calculated for all individual animals. **a** Mouse skeletal muscle vascular endothelial cell activation signature time course. **b** Mouse skeletal muscle mitosis signature time course

Gene expression signatures were established as a set of genes with highly correlated expression profiles belonging to the same molecular pathway or cell type, and manually curated based on peer-reviewed literature analysis (Stiehl et al. 2017). Liver, skin, and muscle were analyzed for gene expression profiles. In liver of both species, there was no observable regulation of gene expression profile (GEP) for VEC activation; however, xenobiotic metabolism induction in hepatocytes was very distinct, with CYP450, UDPGTs, sulfotransferases, acetyltransferases induced from day 1 onward. Specific VEC GEPs in the skin were also difficult to determine due to the large number of dividing cells in this tissue of furred animals. In muscle tissues, clear VEC-specific signatures (Supplementary data 1) in all siponimod-treated groups in both species were identified. In mouse muscle, there was a significant increase of GEP signature of vascular endothelial cell activation from day 1 (5 h post-dose) and sustained over all scheduled sacrifices, with a slow decreasing trend towards the latest time points, however remaining significantly above time-matched controls (Fig. 1a). Similarly, a mitosis gene expression signature (Supplementary data 1) followed a similar pattern but was mostly upregulated from day 3 and sustained over time with a decrease towards later time points (Fig. 1b). There was a statistically significant correlation at all time points from day 3 onward between the vascular endothelial cell activation and mitosis gene expression signatures (*r*^2^ from 0.5 to 0.7). For both gene signatures, there was no dose dependence.

**Fig. 2:**
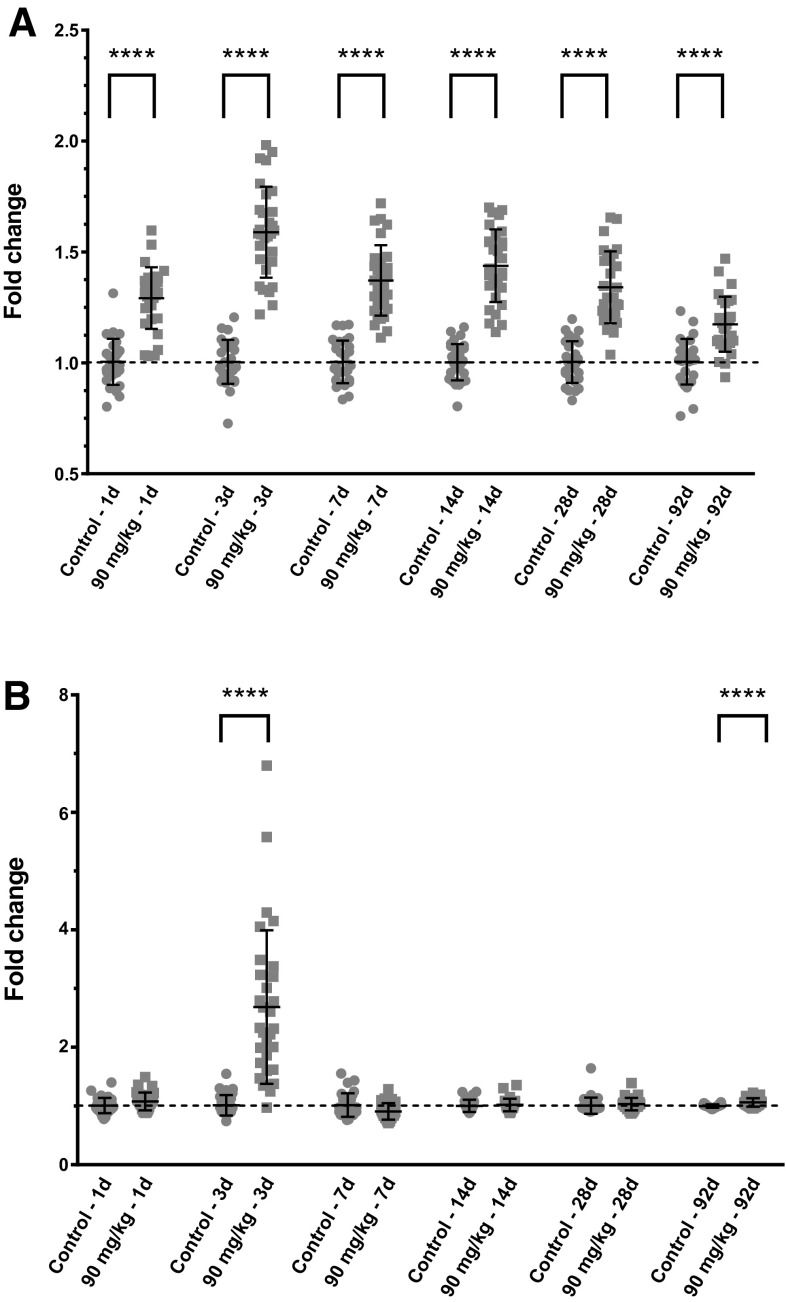
Rat skeletal muscle gene expression. Circle: control, square: high dose. Each dot represents the gene signature of 1 animal. ****p < 0.0001 (homoscedastic *t* test). **a** Rat skeletal muscle vascular endothelial cell activation signature time course. **b** Rat skeletal muscle mitosis signature time course. Each dot represents endothelial vascular cell activation gene signature

In rat muscle, VEC activation GEP was similar to mouse with an upregulation from day 1 and sustained with a slow decrease over time and remaining above controls (Fig. 2a). However, the amplitude of the GEP modulation was notably lower than in the mouse. In addition, in contrast to the mouse, the mitotic GEP pattern in rat was strikingly different with a treatment-related increase only at day 3. All other time points were equal to controls; the statistical significance at day 92 is due to particularly homogeneous control values and is unlikely to reflect a biological effect. This is further supported by the fact that day-92 treated animal values did not show statistical significant differences when compared to the average of all time points controls (Fig. 2b).

In the skeletal muscle of siponimod-treated mice, the endothelial cell progenitor marker CD133 was upregulated, which is normally not expressed in mature endothelial cells (Hristov et al. 2003; Petrenko et al. 1999; Yin et al. 1997), suggesting that local endothelial cell progenitors were activated and were likely the cell population which was proliferating. This was supported by immunohistochemical localization of Ki67-positive cells between muscle fibers in treated mice (Supplementary data 2), with a statistically significant correlation between the mitosis gene expression signature and Ki67-positive cells (*r*^2^ = 0.737) at all time points. In addition, the Ki67-positive cells were co-localized with in situ hybridization detection of CD93-positive cells (Supplementary data 2), a marker of hematopoietic, white blood, and endothelial cells. Hence, in mouse muscle, the cellular proliferation upon siponimod exposure was very likely the vascular endothelial cell compartment. The correlation of immunohistochemical localization of Ki67-positive cells and mitosis gene expression signature in gastrocnemius muscle fibers of treated rat was also statistically significant, with only day 3 being positive for both endpoints.

### Plasma growth factors analysis

**Fig. 3:**
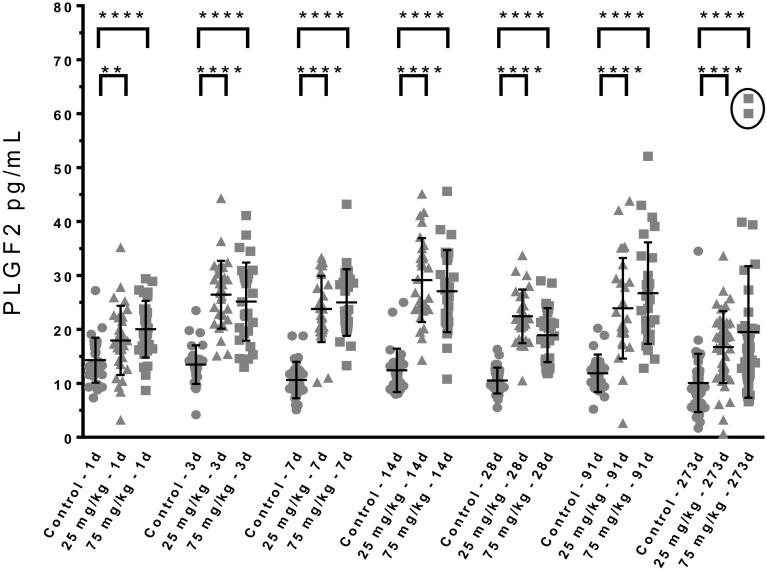
Mouse Placental Growth Factor 2 (PLGF2). Circle: control, triangle: low dose, square: high dose. Each dot represents terminal plasma value of 1 animal. The 2 circled points highlight the 2 mice bearing HSA. **p < 0.01; ****p < 0.0001 (homoscedastic *t* test). Bar = Mean; error bar = 1 standard deviation

A multiplexed analytical method based on magnetic beads and coupled antibodies (Luminex) was applied to mouse plasma samples to analyze concentrations of various circulating pro- and anti-angiogenic factors. In mouse blood, there was an increase of circulating PLGF2 from day 1 that was sustained at all time points without abating (ranging from + 40 to + 135% vs. controls taking into account only statistically significant changes). The 2 animals bearing hemangiosarcoma at the last time point had the highest PLGF2 values (Fig. 3). Of note in control animals, there was a statistically significant decreasing trend of circulating PLGF2 from day 7 over time, presumably a normal decline with age.

**Fig. 4:**
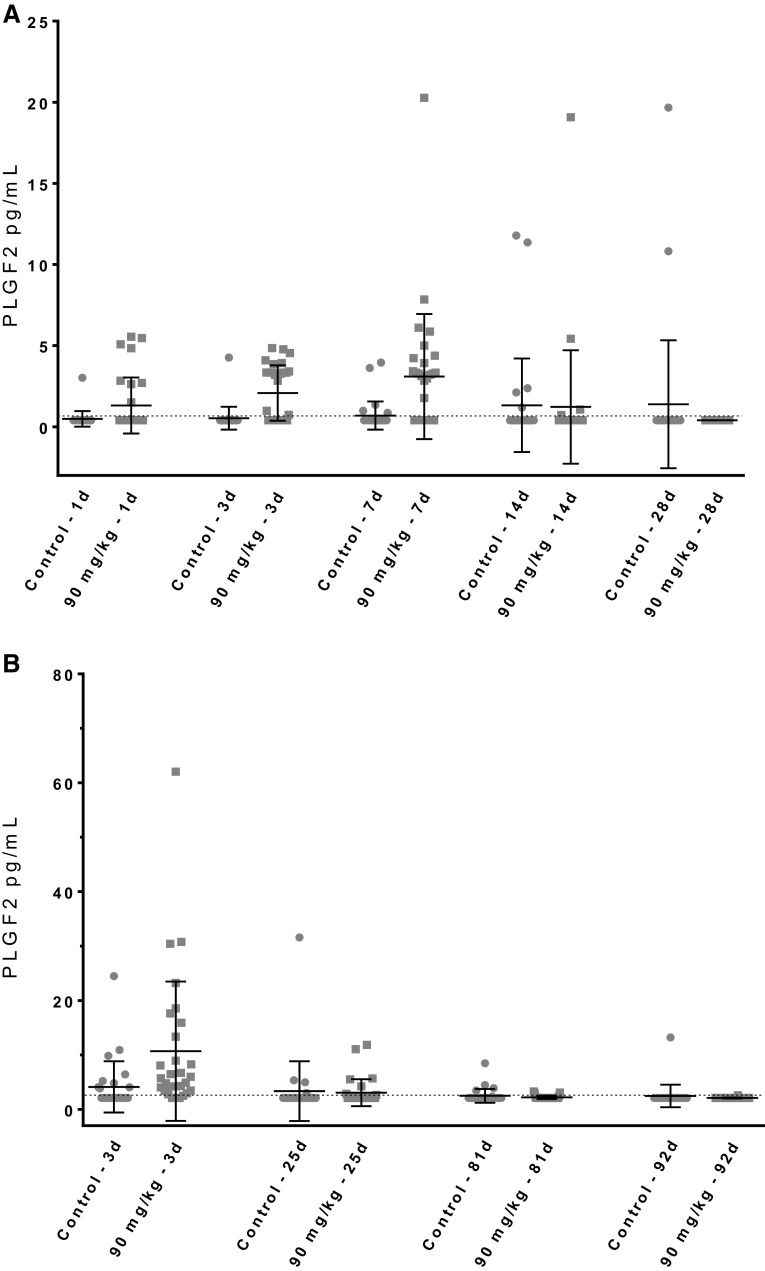
Rat Placental Growth Factor 2 (PLGF2). Circle: control, square: high dose. Bar = mean; error bar = 1 standard deviation. Horizontal-dotted line: LLoQ. Each dot represents terminal plasma value of 1 animal in part A, and same animals intermediate sampling values through time in part B. **a** PLGF2 terminal samples analyzed by Luminex multiplexed kit. **b** PLGF2 kinetics samples analyzed by ELISA

There was no upregulation of PLGF2 mRNA as analyzed by gene array, which is consistent with its described post-translational regulation mechanism (Maglione et al. 1993). Other factors also showed significant increases although to a lesser extent than PLGF2, such as VEGF-C, TNFα and CXCL1, while plasma leptin and soluble endoglin, both anti-angiogenic factors were downregulated (data not shown). Soluble endoglin is known to counteract the pro-angiogenic activity of TGFβ (Levine et al. 2006; Venkatesha et al. 2006). Two different analytical methods were used to measure rat PLGF2, a multiplexed kit developed for mouse PLGF2 applied to terminal samples (Fig. 4a) and a mouse ELISA applied to samples collected at various time points from the group sacrificed at day 92 (Fig. 4b). Many samples were below the LLoQ, which removed the statistical power of the analysis. Nonetheless, the transient nature of this induction was apparent with increases from day 1 to 7 (Fig. 4a, b) when compared to their time-matched controls. Concentrations at later time points were in the range of time-matched control values indicating an initial surge, followed by return to baseline.

### In vitro endothelial vascular cell translational analysis

Cell culture conditions were established for primary CD1 mouse skeletal muscle microvascular endothelial cells to study the effect of siponimod in vitro at concentrations ranging from 1 pM to 30 µM. This nominal concentration range is bracketing siponimod IC_50_ for S1P1 (0.4 nM) and covering a large range of the in vivo plasma and tissue concentration in both species (Table 1). Cell number, cell proliferation monitored by incorporation of EdU into de novo DNA synthesis, and release of PLGF2 protein in media were monitored. The same experiments were conducted on primary Sprague-Dawley rat pulmonary microvascular and aortic endothelial cells, and on primary human dermal and pulmonary microvascular endothelial cells. Although commercially available, rat skeletal muscle microvascular endothelial cells were not used as they did not display all characteristics of VEC, in particular the lack of expression of CD31 (platelet endothelial cell adhesion molecule), von Willebrand factor and S1P1 receptor. Human skeletal muscle VEC were not commercially available.

**Fig. 5:**
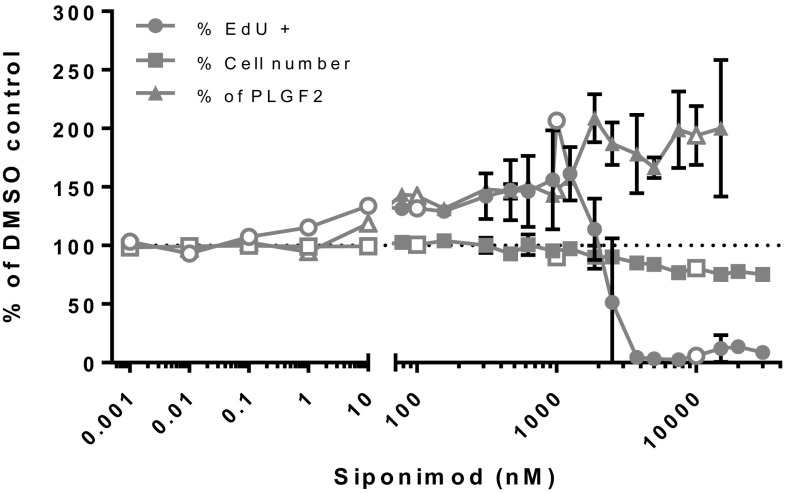
Siponimod-treated mouse skeletal muscle VECs. Assessment of cell proliferation and PLGF2 release of mouse muscle VECs treated with Siponimod expressed as percentage of control untreated cells (solid symbols: average of three independent experiments with triplicate points within each experiment; open symbols: single experiment with triplicate points within each experiment)

The exposure of mouse VECs to sub-pharmacological to mildly cytotoxic concentrations (from 1 pM to 30 µM) of siponimod resulted in an induction of EdU incorporation in cells and PLGF2 release in the medium within its pharmacological range (Fig. 5). Siponimod concentrations above 1 µM led to a sharp decrease of EdU incorporation without cell number decrease, likely revealing a cytostatic effect preceding cytotoxicity. PLGF2 release, with background levels of about 2 µg/mL in controls, was increased at all concentrations ≥ 10 nM siponimod and plateaued up to the highest concentration tested. A mild cytotoxicity effect was observed at ≥ 5 µM. These in vitro experiments were able to replicate the in vivo activation and proliferation of the mouse VECs, as well as the concomitant release of the pro-angiogenic factor PLGF2.

**Fig. 6:**
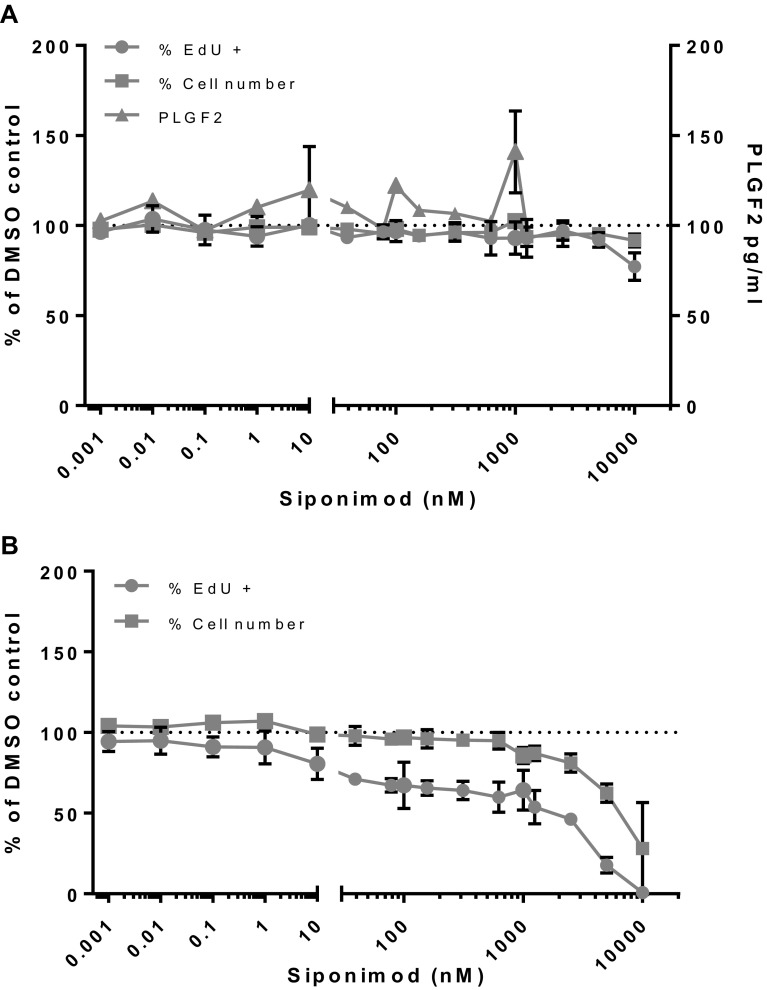
Siponimod-treated rat pulmonary (**a**) and aortic VECs (**b**). Assessment of cell proliferation and PLGF2 release of rat pulmonary (**a**) and aortic (**b**) VECs treated with Siponimod expressed as percentage of control untreated cells (average of two independent experiments with triplicate points within each experiment)

Under the same conditions, exposure of rat pulmonary (Fig. 6a) and rat aortic VECs (Fig. 6b) to siponimod did not induce cell proliferation. PLGF2 levels secreted by untreated pulmonary VEC were near the LLoQ of the assay (93.75 pg/mL). There was a minimal increase in release of the pro-angiogenic factor PLGF2 in an inconsistent concentration-dependent manner, although to a much lesser extent than the mouse VEC cultures. PLGF2 release was not detectable from rat aortic cells. There was a noticeable cytostatic (≥ 10 nM) and cytotoxic (≥ 1 µM) effect of siponimod on the aortic VECs, but not on pulmonary VECs. The rat VEC cultures, conducted under the same conditions as for mouse VECs, mimicked the lack of VEC proliferation in rats observed in vivo. The very striking difference of PLGF2 release in vivo between mouse and rat seems to be reproduced in vitro in VEC cultures.

**Fig. 7:**
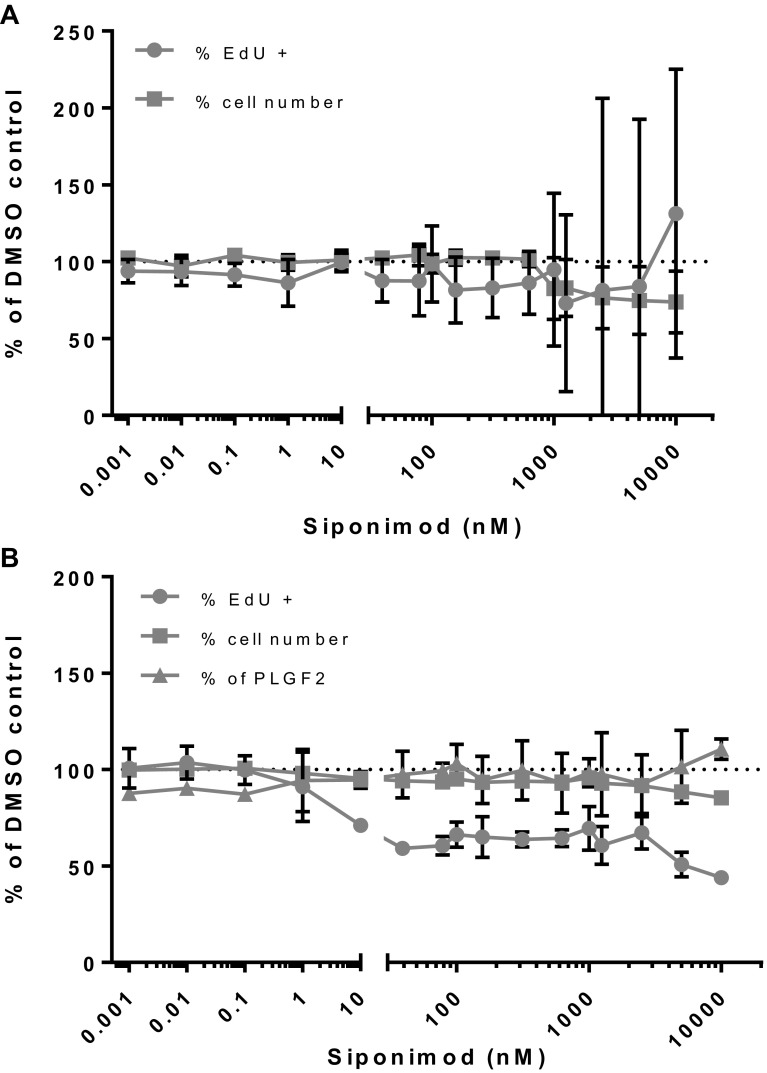
Siponimod-treated human dermal and pulmonary VECs. Assessment of cell proliferation and PLGF2 release of human dermal (**a**) and pulmonary (**b**) VECs treated with Siponimod expressed as percentage of control untreated cells (average of three (pulmonary VECs) and two (dermal VECs) independent experiments with triplicate points within each experiment)

Human pulmonary and dermal VECs under siponimod treatment, similarly to rat VECs were unaffected in terms of proliferation (Fig. 7a, b, respectively). Likewise, baseline PLGF2 concentrations of around 100 pg/mL in controls were also unchanged in response to siponimod. There was a cytostatic effect in pulmonary VECs at ≥ 10 nM without significant cytotoxicity before 10 µM. Quantification of PLGF2 was performed using a validated human kit and all values from human pulmonary VECs were within the linear range of the standard curve and the LLoQ was 15.6 pg/mL, but dermal VECs did not secrete detectable levels of PLGF2. Hence, the human VECs of dermal and pulmonary origin behaved in vitro as the rat pulmonary and aortic VECs, showing no proliferation or changes in release of PLGF2, as opposed to the mouse VECs displaying proliferation and clear release of PLGF2, a specific behavior matching the in vivo observations.

## Discussion

Hemangiosarcoma (HSA) is a malignant tumor arising from the transformation and proliferation of vascular endothelial cells and is a rare tumor in humans with less than 0.001% affected. In rats and mice, the spontaneous incidence is described to be between 0.1 and 2%; and between 2 and 5%, respectively (Cohen et al. 2009). The more common vascular tumors observed in humans are benign hemangiomas and angiomas which are considered separate entities from HSA (Richter and Friedman 2012). Mouse vascular endothelial cells show a high background rate of proliferation and are particularly responsive to pro-angiogenic signals compared to rat and human (Duddy et al. 1999), which is likely related to the high spontaneous rate of HSA in mice (Cohen et al. 2009; Ohnishi et al. 2007).

Activation of angiogenesis does not necessarily lead to abnormal, cancerous blood vessel development; however, some level of angiogenesis is a necessary step toward HSA. In healthy adult individuals, angiogenesis is associated with wound healing and tissue repair (Kawanabe et al. 2007; Lee et al. 2000), and in ovaries and uterus during the female reproductive cycle (Dunlap et al. 2010; Skaznik-Wikiel et al. 2006). In the case of cancerous tissues formation, tumor-induced angiogenesis is an essential feature of tumor growth (Blaho and Hla 2014; Cuvillier et al. 2013; Kunkel et al. 2013; Pyne and Pyne 2010; Rosen et al. 2013; Takabe and Spiegel 2014; Takuwa et al. 2010). Angiogenesis is tightly regulated by a large number of factors and pathways, where vascular endothelial cells, stromal cells (fibroblasts), the extracellular matrix, and pericytes play critical-defined roles (Potente et al. 2011). Several key angiogenic and anti-angiogenic factors such as VEGF-A, VEGF-C, PLGF2, TNFα, CXCL1, and soluble endoglin also contribute to dynamic equilibria (Carmeliet 2005; Cohen 2006).

The present studies were aimed at investigating in vivo mechanisms leading to HSA in the mouse treated with siponimod and their comparative biology and translational relevance for rats in vivo and in vitro, and humans in vitro. In the mouse, daily oral administration of siponimod-induced VEC activation, and secretion of placental growth factor 2 (PLGF2) were followed by induction of cell proliferation. These effects were observed immediately and persisted throughout the 9 months of treatment. The location and morphology of the CD93-positive VECs in mouse skeletal muscle co-localized with the proliferative marker Ki67 staining, compatible with a vascular endothelial cell (VEC) origin thereby linking the HSA formation with VEC proliferation.

PLGF2 is known to be mostly produced by vascular endothelial cells in adults (De Falco 2012) and despite a clear increase in circulating PLGF2; there was no transcriptional upregulation of its mRNA in the studied tissues. This is in agreement with its described regulation by post-transcriptional modulation independent of mRNA levels, as described for a number of other growth factors (De Falco 2012). The PLGF2 mRNA contains a *cis*–*trans* regulatory sequence upstream of the coding region, acting as a repressor of the mRNA translation that has been shown to be the mechanism by which PLGF2 protein level is regulated (Maglione et al. 1993).

PLGF2 is involved during the same phases and steps of angiogenesis as S1P (Kunkel et al. 2013; Odorisio et al. 2002; Ziche et al. 1997). Soluble PLGF2 was increased from day 1, as early as 5 h after the 1st oral dose administration of siponimod, suggesting that it is an immediate response to S1P receptor modulation in VECs. PLGF2 is a powerful pro-angiogenic growth factor which is normally expressed only during embryogenesis, wound healing and in ovaries during the female reproductive cycle. It is also induced during neo-angiogenesis in solid tumor vascularization (Odorisio et al. 2002; Ziche et al. 1997). In addition, PLGF2 over-expression on its own has been shown to impact vascularization in adult mice: increased vessel number, tortuosity and size, vessel spike emission, intussusception and glomeruloid bodies (Odorisio et al. 2002). On the other hand, PLGF2 knock-out mice are normal and fertile (Carmeliet and Jain 2011), which demonstrated its redundancy for vascular development and physiological vessel maintenance in healthy adult mice (De Falco 2012). Due to its role in tumorigenesis, it is possible that the high levels of PLGF2 seen in the two HSA-bearing mice might have resulted from the combination of the drug-induced release, and perhaps secretion from the vascular tumor cells themselves. This potentially synergistic effect might be involved, at least partly, in the observed sudden burst of HSA formation in the 2-year carcinogenicity study whereby the first tumors appeared at around 9 months of treatment with a peak of HSA-linked premature death in the months 12–18. This fast onset may lead to a very narrow time-window where pre-neoplastic vascular lesions could be detected, and may explain why none were observed in any tissue in either the carcinogenicity study or the present 9-month investigative study in mice. Consistently, no gene expression signatures characteristic of pre-neoplastic lesions were found upregulated.

**Fig. 8:**
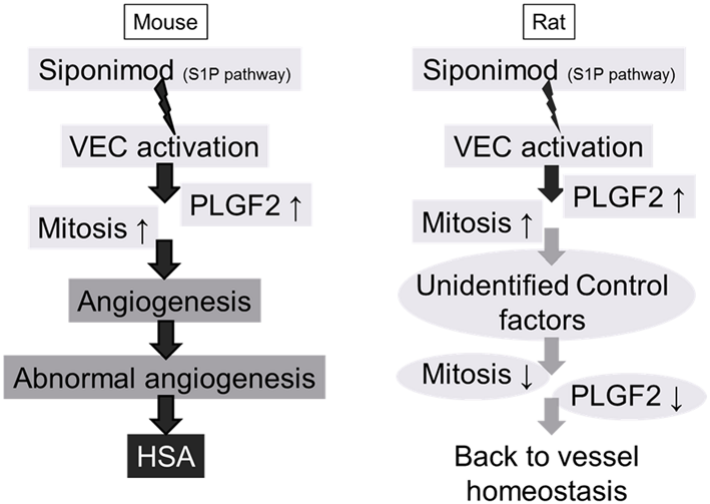
Mouse versus rat Adverse Outcome Pathway plausible mechanism for HSA formation in the mouse. Although PLGF2 induction was observed earlier than the mitosis gene signature, it is unknown if one flows from the other, or if both independently arise from VEC activation. The fact that in the rat, PLGF2 induction outlasts mitosis would tend to advocate for two separate events

In the mouse, the early and sustained VEC activation and secretion of PLGF2, followed by mitosis induction, likely led to activated neo-angiogenesis which over life-long treatment may drive VEC proliferation and finally HSA (Fig. 8). In rats, although VECs were activated throughout the duration of the study, the angiogenic mitosis signature and increased PLGF2 production were only transient during the first week, both returning to control values after an initial surge. Therefore, for both HSA occurrence and molecular mechanisms relevance, the 3-month duration chosen for the rat mechanistic study appeared adequate and the molecular mechanisms at stake are already firmly in place after such a treatment duration. In addition, since after 2 years of treatment in the carcinogenicity study, rats did not develop HSA, it did not appear necessary to prolong the treatment beyond 3 months, and it would have been unethical to extend this study which can already be considered as chronic. Despite the constant VEC activation in rats which was also likely the consequence of siponimod modulation of S1P1 receptor, the absence of sustained mitosis and angiogenic factors did not result in the formation of HSA (Fig. 8). A similar divergence between mouse and rat has previously been described for Pregabalin where a sustained hypoxia in the mouse, which is only transient in the rat, had induced hemangiosarcoma in mice, but not in rats or in humans (Cohen et al. 2009; Pegg et al. 2012). This hypoxia mechanism has also been proposed for PPARγ agonists like Troglitazone and for a chemical, 2-butoxyethanol, where the hypoxia-inducible factor 1 (HIF-1) plays a critical role (Cohen et al. 2009). HIF-1 is known to be a key transcription factor induced in case of hypoxic conditions, which regulates a large number of gene transcription involved in several pathways, including aspects of cancer biology, angiogenesis, cell survival and invasion, and glucose metabolism (Semenza 2003). In the present case, an induction of hypoxia/HIF-1 gene expression profile for HIF-1 itself and known downstream gene targets was not observed in mice or rats. On the contrary, the observed PLGF2 induction would tend to support a downregulation of HIF-1, since hypoxia has been shown to downregulate PLGF2 in human in vivo and that hyperoxia upregulates PLGF2 in vitro in cell culture (Khaliq et al. 1999). Hence, in the present case, HSA in the mouse appears to be induced by a different mechanism than hypoxia. However, consistent with these prior descriptions of hypoxia/HIF1-induced HSA in the mouse, a chronic and sustained response was unique to the mouse and was not translatable to rat or human.

The mouse and rat endothelial primary cell cultures also demonstrated different responses upon exposure to siponimod, matching their respective in vivo responses. Mouse cell proliferation within a pharmacological dose range of siponimod in the absence of cytotoxicity was sensitively demonstrated by incorporation of EdU into de novo DNA synthesis. PLGF2 release paralleled cell proliferation at low concentrations, and remained elevated at higher concentrations, seemingly not affected by the cytostatic effect of siponimod at high concentrations. The presence of PLGF2 and its induction by siponimod further support that in vivo, the observed increase of this circulating growth factor is coming from the VECs, despite the absence of transcriptional upregulation. This is also consistent with the translational regulation mechanism described for PLGF2 (De Falco 2012).

In contrast, rat cells were insensitive to siponimod treatment up to 5 µM in the absence of cell death, displaying no cell proliferation. The basal release of PLGF2 in media from control cells was about 20-fold lower for the rat than for the mouse. There was an equivocal increase in PLGF2 content in media upon treatment in rat cell cultures, but considerably lower than that of the mouse cultures, mirroring the respective in vivo observations. Human VECs of dermal and pulmonary origins demonstrated an absence of proliferative response analogous to rat cells in vitro, and did not show siponimod-induced release of PLGF2 at all tested concentrations from 1 pM to 10 µM. The amount of the spontaneous PLGF2 release of untreated human dermal VECs was very similar to those of the rat. Since the mouse and rat cells in vitro mirrored their respective cell proliferation and PGLF2 release in vivo profile, we hypothesize that the human cells would also reflect a lack of in vivo response to siponimod. This position is further supported by the Pregabalin precedent where the mouse HSA induction did not translate to man and is not identified as an adverse drug reaction (Fuzier et al. 2013). Therefore, HSA formation in the mouse model of carcinogenicity appears not predictive to humans, both in terms of spontaneous occurrence (2–5% in mice against 0.001% in human) (Cohen et al. 2009), as well as for drug-induced HSA incidence (Kakiuchi-Kiyota et al. 2009). We conclude that the molecular mechanisms leading to siponimod-induced hemangiosarcoma in mice are considered species specific and are likely irrelevant to human.

## Electronic supplementary material

Below is the link to the electronic supplementary material.

Supplementary material 1 (DOCX 20 KB)

Supplementary material 2 (DOCX 1352 KB)
