# Supplementary material for: Induction of hemangiosarcoma in mice after chronic treatment with S1P-modulator siponimod and its lack of relevance to rat and human
Source: Arch Toxicol. 2018 Mar 19;92(5):1877–91. doi: 10.1007/s00204-018-2189-9 (PMC5962627; doi:10.1007/s00204-018-2189-9)
Supplement: Supplementary file 1 — Supplementary material 1 (DOCX 20 KB) [file 204_2018_2189_MOESM1_ESM.docx]

**Supplementary data 1**

Mouse Endothelial cell activation Gene signature

| **Probe Set** | **Gene Symbol** | **Gene Title** |
| --- | --- | --- |
| 1416077_at | Adm | adrenomedullin |
| 1418421_at | Bcl6b | B cell CLL/lymphoma 6, member B |
| 1456046_at | Cd93 | CD93 antigen |
| 1417839_at | Cldn5 | claudin 5 |
| 1437308_s_at | F2r | coagulation factor II (thrombin) receptor |
| 1422445_at | Itga6 | integrin alpha 6 |
| 1424067_at | Icam1 | intercellular adhesion molecule 1 |
| 1448862_at | Icam2 | intercellular adhesion molecule 2 |
| 1416295_a_at | Il2rg | interleukin 2 receptor, gamma chain |
| 1452514_a_at | Kit | kit oncogene |
| 1417595_at | Meox1 | mesenchyme homeobox 1 |
| 1456768_a_at | Mmrn2 | multimerin 2 |
| 1439389_s_at | Myadm | myeloid-associated differentiation marker |
| 1435349_at | Nrp2 | neuropilin 2 |
| 1421287_a_at | Pecam1 | platelet/endothelial cell adhesion molecule 1 |
| 1425681_a_at | Prn | prion protein gene complex |
| 1449135_at | Sox18 | SRY (sex determining region Y)-box 18 |
| 1433575_at | Sox4 | SRY (sex determining region Y)-box 4 |
| 1416564_at | Sox7 | SRY (sex determining region Y)-box 7 |
| 1415989_at | Vcam1 | vascular cell adhesion molecule 1 |

Mouse Mitosis signature

| **Probe Set** | **Gene Symbol** | **Gene Title** |
| --- | --- | --- |
| 1426817_at | Mki67 | antigen identified by monoclonal antibody Ki 67 |
| 1439377_x_at | Cdc20 | cell division cycle 20 |
| 1423682_a_at | Cdca4 | cell division cycle associated 4 |
| 1424143_a_at | Cdt1 | chromatin licensing and DNA replication factor 1 |
| 1416873_a_at | Cdk2 | cyclin-dependent kinase 2 |
| 1438009_at | Hist1h2ab | histone cluster 1, H2ab |
| 1439436_x_at | Incenp | inner centromere protein |
| 1437716_x_at | Kif22 | kinesin family member 22 |
| 1420028_s_at | Mcm3 | minichromosome maintenance deficient 3 (S. cerevisiae) |
| 1436808_x_at | Mcm5 | minichromosome maintenance deficient 5, cell division cycle 46 (S. cerevisiae) |
| 1416251_at | Mcm6 | minichromosome maintenance deficient 6 (MIS5 homolog, S. pombe) (S. cerevisiae) |
| 1428483_a_at | Mis18a | MIS18 kinetochore protein homolog A (S. pombe) |
| 1434437_x_at | Rrm2 | ribonucleotide reductase M2 |
| 1454656_at | Spata13 | spermatogenesis associated 13 |

Rat Endothelial cell activation Gene signature

| **Probe Set** | **Gene Symbol** | **Gene Title** |
| --- | --- | --- |
| 1387219_at | Adm | adrenomedullin |
| 1381804_at | Bcl6b | B-cell CLL/lymphoma 6, member B |
| 1386832_a_at | Bcl6b | B-cell CLL/lymphoma 6, member B |
| 1386833_at | Bcl6b | B-cell CLL/lymphoma 6, member B |
| 1394375_x_at | Bcl6b | B-cell CLL/lymphoma 6, member B |
| 1368393_at | Cd93 | CD93 molecule |
| 1387168_at | Cd93 | CD93 molecule |
| 1374104_at | Cldn5 | claudin 5 |
| 1367899_at | F2r | coagulation factor II (thrombin) receptor |
| 1371185_at | Itga6 | integrin, alpha 6 |
| 1383240_at | Itga6 | integrin, alpha 6 |
| 1393558_at | Itga6 | integrin, alpha 6 |
| 1387202_at | Icam1 | intercellular adhesion molecule 1 |
| 1389235_at | Icam2 | intercellular adhesion molecule 2 |
| 1390310_at | Icam2 | intercellular adhesion molecule 2 |
| 1389092_at | Il2rg | interleukin 2 receptor, gamma |
| 1393974_at | Meox1 | mesenchyme homeobox 1 |
| 1375966_at | Mmrn2 | multimerin 2 |
| 1388453_at | Myadm | myeloid-associated differentiation marker |
| 1369824_at | Nrp2 | neuropilin 2 |
| 1382995_at | Nrp2 | neuropilin 2 |
| 1371545_at | Pecam1 | platelet/endothelial cell adhesion molecule 1 |
| 1384924_at | Prnd | prion protein 2 (dublet) |
| 1368008_at | Prom1 | prominin 1 |
| 1381971_at | Sox18 | SRY (sex determining region Y)-box 18 |
| 1373860_at | Sox4 | SRY (sex determining region Y)-box 4 |
| 1375123_at | Sox4 | SRY (sex determining region Y)-box 4 |
| 1383137_at | Sox4 | SRY (sex determining region Y)-box 4 |
| 1383989_at | Sox4 | SRY (sex determining region Y)-box 4 |
| 1384000_at | Sox4 | SRY (sex determining region Y)-box 4 |
| 1393571_at | Sox4 | SRY (sex determining region Y)-box 4 |
| 1384415_at | Sox7 | SRY (sex determining region Y)-box 7 |
| 1368474_at | LOC100912479 | vascular cell adhesion protein 1-like |
| 1369822_at | Kit | v-kit Hardy-Zuckerman 4 feline sarcoma viral oncogene homolog |
| 1376648_at | Mycn | v-myc avian myelocytomatosis viral oncogene neuroblastoma derived homolog |
| 1387001_at | Ralb | v-ral simian leukemia viral oncogene homolog B |

Rat Mitosis signature

| **Probe Set** | **Gene Symbol** | **Gene Title** |
| --- | --- | --- |
| 1393581_at | Aspm | asp (abnormal spindle) homolog, microcephaly associated (Drosophila) |
| 1373823_at | Cks2 | CDC28 protein kinase regulatory subunit 2 |
| 1379582_a_at | Ccna2 | cyclin A2 |
| 1389566_at | Ccnb2 | cyclin B2 |
| 1367776_at | Cdk1 | cyclin-dependent kinase 1 |
| 1367676_at | Hmgb2 | high mobility group box 2 |
| 1374775_at | Mki67 | marker of proliferation Ki-67 |
| 1373658_at | Racgap1 | Rac GTPase-activating protein 1 |
| 1389408_at | LOC100359539 | ribonucleotide reductase M2 polypeptide |
| 1388650_at | Top2a | topoisomerase (DNA) II alpha |
