# Supplementary material for: Induction of hemangiosarcoma in mice after chronic treatment with S1P-modulator siponimod and its lack of relevance to rat and human
Source: Arch Toxicol. 2018 Mar 19;92(5):1877–91. doi: 10.1007/s00204-018-2189-9 (PMC5962627; doi:10.1007/s00204-018-2189-9)
Supplement: Supplementary file 2 — Supplementary material 2 (DOCX 1352 KB) [file 204_2018_2189_MOESM2_ESM.docx]

**Supplementary data 2**

Comparison of endothelial cell staining (CD31) to angiogenic (CD93) and proliferation (Ki67) staining pattern in the muscle. Similar staining pattern of CD31 in animals treated with vehicle or BAF312 at day 3 (column A, arrows). Increased CD93- and Ki67-positive cells between the muscle fibers in BAF312-treated mice after 3 days (column B and C, arrows).

**A B C**


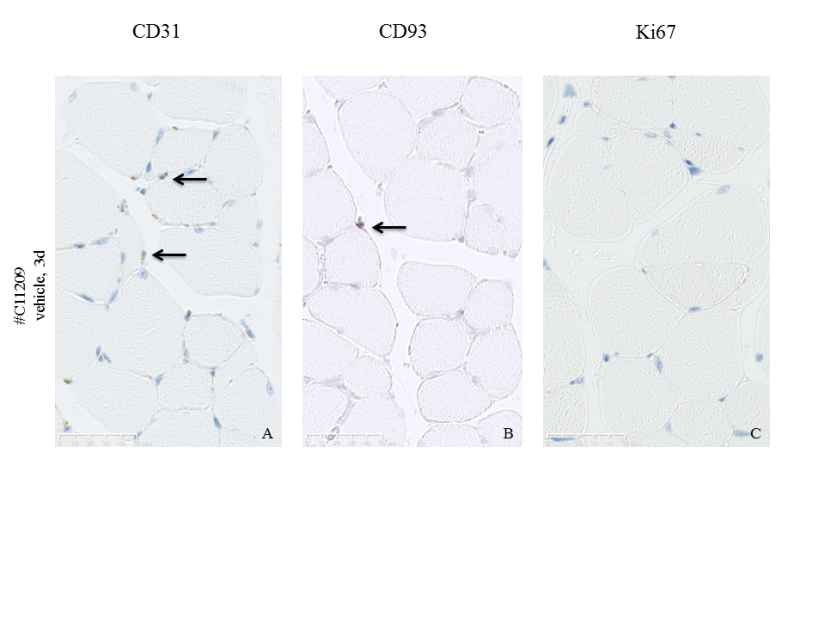


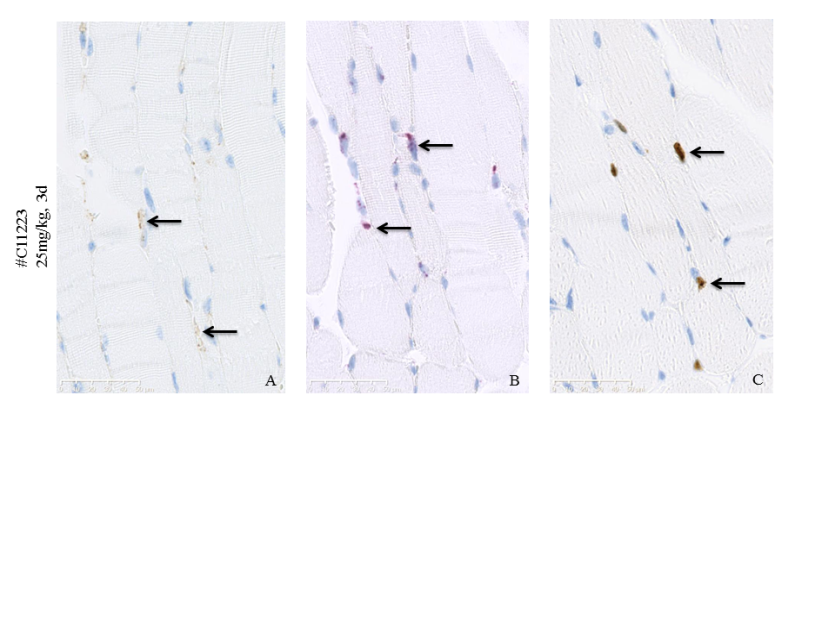


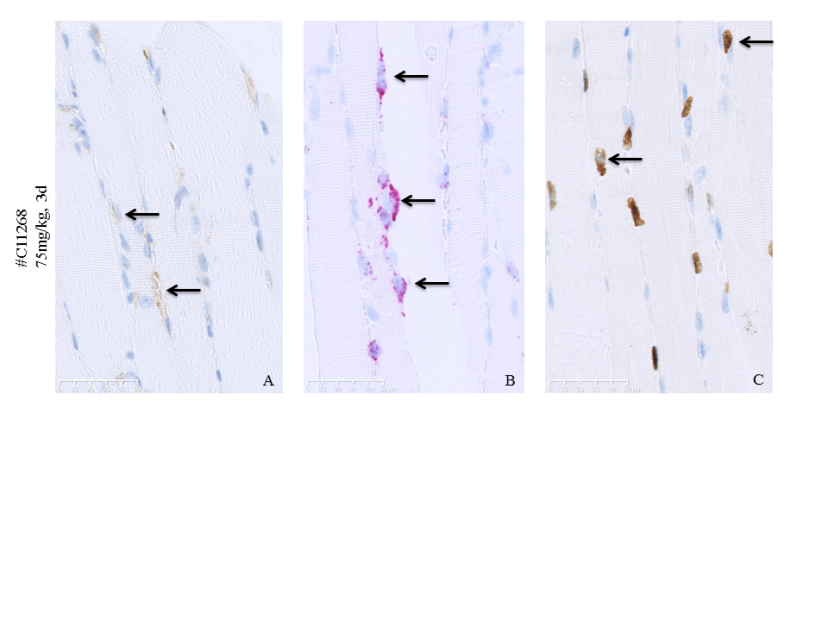


Correlation of relative number of Ki67 positive nuclei versus Mitosis gene signature in mouse muscle.

"Mitosis" Gene Signature Score, Log2

Ki67 positive per area
